# Challenges in sexual and reproductive healthcare access for queer people in Gauteng, South Africa

**DOI:** 10.4102/phcfm.v16i1.4726

**Published:** 2024-11-29

**Authors:** Raikane J. Seretlo, Hanlie Smuts, Mathildah M. Mokgatle

**Affiliations:** 1 Department of Public Health, School of Health Sciences, Sefako Makgatho Health Sciences University, Tshwane, South Africa; 2 Department of Informatics, Faculty of Engineering, Built Environment and Information Technology, University of Pretoria, Tshwane, South Africa

**Keywords:** LGBT, challenges, queer people, healthcare professionals, sexual and reproductive healthcare services

## Abstract

**Background:**

Sexual and reproductive healthcare services and needs (SRHS) are essential for improving overall health and well-being. Queer people face obstacles not only in obtaining and receiving SRHS but also in the provision of these services by healthcare professionals (HCPs).

**Aim:**

The study explored and described the challenges experienced by HCPs and queer people when providing and accessing SRHS in the Gauteng province.

**Setting:**

We collected data from seven district public hospitals and two non-governmental organisation clinics that focussed on queer-related healthcare in the Gauteng province, South Africa.

**Methods:**

This was an explorative-descriptive qualitative study, in which we conducted 55 one-on-one interviews. The researchers employed purposive sampling to select all HCPs, and respondent-driven sampling for all queer people who participated in the study.

**Results:**

Six main themes emerged, demonstrating that HCPs and queer people faced similar, contrasting and differing challenges when rendering and receiving SRHS. These themes include HCPs’ belief that queer people are afraid, while queer people perceive HCPs as having negative attitudes and acting as gatekeepers. HCPs expressed surprise and confusion regarding gender identity, healthcare disparities and familial issues, which highlighted their feelings of incompetence in providing queer-related healthcare and their engagement with queer people as a barrier.

**Conclusion:**

Policies need to be strengthened to ensure inclusivity in the healthcare sector, thus, addressing SRHS for queer people.

**Contribution:**

The findings from this study have the potential to bridge discrepancies and address the challenges faced by both HCPs and queer people through policy development.

## Introduction

Sexual and reproductive health (SRH) refers to in-depth comprehension of a person’s physical and mental well-being regarding sexuality and reproduction.^1^ This comprises not only a lack of sickness or disability, but also a condition of complete physical, mental and social well-being in all aspects of the reproductive system.^1^ It entails having the capacity for a safe and fulfilling sexual life, the capacity to reproduce and the discretion to choose whether, when and how frequently to do so.^1^ Oringanje et al.^2^ suggest preventing unwanted pregnancies by motivating teenagers to delay becoming sexually active, promoting contraception and teaching girls and boys about the dangers. According to the National Academies of Sciences,^3^ SRH is an important framework for preventing and controlling sexual transmitted infections (STIs). These involve the prevention and control of STIs such as chlamydia, which has significant consequences for future fertility.^4^

Therefore, everyone should have access to high-quality vital healthcare as the cornerstone of universal health coverage.^5^ However, adolescents across the world continue to experience SRH challenges, such as early pregnancy and parenthood, difficulties accessing contraception and safe abortion, and high rates of human immunodeficiency virus (HIV) and STIs.^6^ In addition, Blanco^7^ discovered that culture and ethnicity, socioeconomic status, customs, faith, spoken language, research, education, equality and quality challenges, and policy-direction obstacles influenced global reproductive health programmes and service shortcomings.

In African countries, Melesse et al.^8^ found significant inequities and inconsistent growth across several key adolescent sexual and reproductive health (ASRH) metrics. Furthermore, African countries bear the heaviest burden of HIV infection worldwide, with women accounting for more than 57% of those afflicted.^9^ Women with HIV in sub-Saharan Africa (SSA) have a greater risk of unwanted pregnancy and unsafe abortion behaviours.^9^ A Ugandan study found that potential barriers to HIV–SRH integration comprise patient-provider, such as a lack of training and negative attitudes; universal healthcare system difficulties, such as inadequate staffing; and inadequate facilities with insufficient space and privacy for providing these services.^10^

South African teenagers are in danger of contracting HIV and STIs and becoming pregnant because of high-risk sexual conduct, physical, social and structural barriers, and insufficient availability of critical primary SRH services.^11^ Other young South African women continue to be vulnerable to high rates of teenage pregnancies, HIV, STIs and female genital schistosomiasis (FGS).^12^ Similarly, Ramkissoon et al.^13^ state that South Africans continue to encounter barriers to receiving comprehensive treatment, avoiding and managing STIs, SRH, family planning, pregnancy and delivery and psychological assistance.

In relation to queer people, several studies have stated their specific sexual-reproductive services and needs. For example, several studies have shown the importance of the use of lubricants among queer people during sexual intercourse.^14,15,16,17^ Availability of adoption services within healthcare facilities is noted as some of the important SRH needs and services that queer people normally consider.^18^ Again, gender-affirmation services such as hormones and surgery are identified as some of the important sexual health services for queer people.^17,19,20,21^ Additionally, Hadj-Moussa et al.^22^ stated that gender affirmation services improve the quality of life, happiness and sexual function of transgender people. Other studies emphasised the need for gender-neutral bathrooms as essential sexual and reproductive services for queer people.^17,23,24,25^ However, the findings from Seretlo et al.^17^ showed that queer people require holistic sexual-reproductive services and needs such as the choice to terminate pregnancy, contraceptive accessibility and promotion, services related to HIV, pre-prophylaxis (PrEP) access and STI treatment. Furthermore, Seretlo et al.^17^ stated that breast examinations, male circumcision, pap smears, prostate services and vasectomy services are some of the vital sexual and reproductive services for queer people. Additionally, Herriges et al.^26^ conducted a study on the relationship between sexual orientation and prostate, breast, and cervical cancer screening and diagnoses. All these studies have pinpointed all the important sexual and reproductive services and needs of queer people.

Societies globally still need to do much more because queer people are still experiencing and facing exclusions and a series of health inequities derived from stigmatisation and discrimination in the healthcare sector.^27,28,29,30^ Queer is an abbreviated term for gays, lesbians and others with non-normative sexual identities.^31^ For this article, the word ‘queer’ will describe someone who does not identify as heterosexual or cisgender. Müller^32^ highlights that queer people continue to experience marginalisation both within healthcare systems and their communities as a result of heteronormative and cisnormative systems. These systems inhibit access to healthcare services and perpetuate stigma and discrimination by shaping the expression and understanding of gender and sexuality.^33,34^ By focussing on queer theory and intersectionality, the existing literature highlights how interconnected identities (such as race, socioeconomic status and sexual orientation) exacerbate discrimination against queer people, compromising their healthcare experiences.^35^

Over the past few years, there has been an increased focus on addressing the health-related concerns of gender and sexual minorities, notably queer people. These identities are complicated and fluid, necessitating critical interaction with definitions of gender, sexuality and queerness in order to avoid strict or too simplified views. Researchers like Butler^36^ and Fausto-Sterling^37^ have challenged binary conceptions of gender and sexuality, highlighting how they are constructed by society and performed.

In South Africa, such identities connect with distinct cultural, legal and healthcare frameworks, which have a profound impact on how queer people access and use healthcare. South Africa’s Constitution, known for its liberal human rights approach, enshrines the right to equality and non-discrimination in Section 9, which provides full protection based on gender, sexual orientation and other identities.^38^ This legal base is further strengthened by the *National Health Act* (61 of 2003) and the *Promotion of Equality and Prevention of Unfair Discrimination Act* (PEPUDA, Act 4 of 2000), both of which require equitable access to healthcare services for all people.^39^ Furthermore, the South African National LGBTI HIV Plan (2017–2022) emphasises these difficulties, citing an increased vulnerability to HIV among queer people and stressing the institutional hurdles that prevent fair access to healthcare.^40^ Furthermore, the implementation of National Health Insurance (NHI) seeks to achieve universal health coverage by tackling systematic disparities in healthcare accessibility.^41^

These inequities persist despite the United Nations Sustainable Development Goals (SDGs) seeking to address issues of inequality, embrace diversity and ensure health and well-being for all.^42^ Furthermore, these SDGs, notably SDG 3.7, seek to provide universal access to sexual and reproductive healthcare services by 2030.^42^ In South Africa specifically, queer people continue to face difficult and diverse social challenges, such as prejudice, stigmatisation, denial of healthcare services and community rejection.^32,43,44,45,46,47,48^ There are some huge inconsistencies in the implementations of the current rules, laws and policies. This disconnection combined with social stigma, economic disparities and geographic limitations, highlights the multifaceted nature of healthcare challenges for queer people in the Gauteng province, revealing larger the intersections between identities and systemic unequal treatment.^49^ Trans women, in particular, encounter significant barriers because of a culture of medical genderism, which marginalises their identities and needs.^50^ Furthermore, the heteronormative framework of South Africa’s health system often overlooks the unique experiences of queer people, leading to inadequate service provision.^51,52^

There is still a belief that healthcare professionals (HCPs) are the most crucial frontline workers who can help queer people overcome difficulties. Nevertheless, some HCPs continue to discriminate against queer people based on their sexual orientation or gender identity, expressing moral judgement and disapproval^30,44,53^ and imposing heteronormative attitudes.^54^ Some HCPs refuse to provide care to queer people,^44^ and others lack information about queer people’s identities and health concerns, resulting in poor-quality care.^44,53^ Furthermore, the evidence highlights the impact of HCPs, policy gaps and social views in either facilitating or impeding access to care.^33,55^ Many HCPs in South Africa lack training in queer people-affirming practices, resulting in insensitive or inadequate treatment of queer people.^56^

These issues around queer people are not only prevalent in South Africa but also across continents and countries. For example, around Africa, queer people continue to face stigmatisation and discrimination, which jeopardises their health and exacerbates STIs such as the HIV epidemic.^57^ Furthermore, a study by Mbeda et al.^58^ found that queer people reported experiencing healthcare-related stigmatising experiences, which led to a fear of seeking or an avoidance of healthcare services, and some reported being denied health services because they were queer people. Rosee et al.^59^ found stigmatisation and prejudice as some barriers to healthcare for queer people around Africa, which were exacerbated by the illegality of homosexuality in the past. Furthermore, barriers to HIV testing resulted in the exclusion of some queer people from treatment, a cascade of events and poor sexuality training among HCPs leading to discrimination or denial of treatment for some queer people, noticeably in the government institutions.^59^

Moreover, most countries (aside from South Africa), regardless of their level of healthcare advancement and being classified as developed countries, still have challenges in improving queers’ health and well-being. According to a study by Gonzales and Henning-Smith,^60^ some queer people like transgender and nonconforming adults continue encountering barriers to healthcare because of discrimination, insurance coverage, employment and public policy, as well as an inadequate level of understanding among HCPs about transgender-related health issues. According to Hafeez et al.,^61^ queer people receive low-quality care because of stigmatisation, a lack of knowledge among HCPs and insensitivity to their community’s particular needs. Westwood et al.^62^ discovered nurses’ negative attitudes towards queer people by uncovering a toxic nursing culture in a geriatric ward, and the person involved in this case study described multiple instances, including homophobic and transphobic practices, as well as queer microaggressions, which evidently affected the quality of nursing care. Elliot et al.^63^ found that queer people have poorer health and bad healthcare experiences. Furthermore, systematic review findings by Alencar et al.^54^ indicated that queer people face problems in accessing health services as a consequence of heteronormative views enforced by HCPs.

The obstacles queer people face must be addressed expeditiously to reduce health risks. Unless these issues are addressed, queer people will continue to be uncomfortable with announcing their identities and conceal signs of STIs,^59^ thereby contributing to an escalating HIV epidemic.^57^ Other health challenges from which queer people continue to suffer are a greater likelihood of substance use, STIs, physical malignancies, heart disease, obesity, bullying, separation, disapproval, anxiety, depression and suicide compared to the general population.^61^ Furthermore, they will keep experiencing depression and anxiety as a result of HCPs’ homophobic comments,^64^ and HCPs will continue imposing heteronormative attitudes.^54^ Few studies have focussed on the challenges both queer people and HCPs experience in South Africa. This study significantly enhances our understanding of the healthcare inequities experienced by both queer people and HCPs. By exploring any deficits in understanding, this article aims to explore the challenges queer people and HCPs face when receiving and providing sexual and reproductive healthcare services in South Africa, establishing the foundation for specific measures and changes in policy that promote inclusive, non-discriminatory healthcare practices and, ultimately, enhance the overall well-being of South African queer populations.

## Methods

### Study design

An explorative-descriptive qualitative study was performed to explore and describe the challenges experienced by HCPs during provision of and queer people when accessing SRHS in the Gauteng province, South Africa.

### Study settings

The study was conducted in two main study settings based on the population, that is, for HCPs was the sevel selected government district hospitals around Gauteng province were included and for queer people selected one non-governmental organisation (NGO). To be specific, seven selected public district hospitals and two branches of one NGO were utilised to collect data.

The Gauteng province is one of South Africa’s major provinces and it is considered the smallest province in South Africa (Gauteng Municipalities Web, 2023). The province has five districts, namely, Ekurhuleni, Johannesburg, Sedibeng, Tshwane and West Rand district.^65^ Each district has its own hospitals and clinics. District hospitals fall under the secondary level of care and provide healthcare services for their people. The services that district hospitals usually render are family medicine, paediatrics, obstetrics and gynaecology, emergency, outpatients, operating theatre and allied units such as physiotherapy, occupational therapy and social work, and psychology.^66^ The hospitals operate 24 h; however, some services close at 16:00.^66^

For easy access to queer individuals, the study was conducted around one NGO which renders free queer SRH services in Tshwane. The selected NGO clinic is a facility that offers free sexual health services for gay, bisexual, queer individuals and men who have sex with men (MSM).^67^ This clinic has collaborated with the Aurum Institute, and it operates in five districts across South Africa: Tembisa, Mbombela, Pietermaritzburg, Durban and Pretoria.^67^ The sexual health services offered include counselling, screening for HIV, STIs and tuberculosis (TB), receiving PrEP or antiretroviral therapy (ART), and psychosocial support services inclusive of gender-affirming healthcare and hormonal therapy.^67^ The clinic operates from 08:30 to 16:30. The study focussed only on two branches, namely Tembisa and Pretoria because other districts are outside Gauteng, and therefore, they do not meet the inclusion criteria.

### Population and sampling strategy

The study involved 55 participants (33 HCPs and 22 queer people). Participants in the study comprised nurses, medical physicians, social workers, clinical psychologists (HCPs), lesbians, homosexuals, bisexuals, transgender people and MSM. The study utilised purposive sampling to select 33 HCPs by selecting participants who were knowledgeable about the matter of SRHS and used respondent-driven sampling (RDS) to select 22 queer people as it was difficult to find the selected participants.

### Inclusion criteria

All HCPs who were working at the selected seven district hospitals in the Gauteng province were allowed to take part in the study. HCPs were supposed to be working specifically at the hospital’s gateway clinics, casualty, outpatient departments, obstetric departments, counselling and reproductive health clinics as most of them are the ones coming in contact with queer individuals and render SRHS. All queer people who are 18 years and above in the selected study settings will be included.

### Exclusion criteria

All other HCPs who were working at the different wards (not gateway clinics, casualty, outpatient departments, obstetric departments, pharmacy and reproductive health clinics) at the selected seven district hospitals in the Gauteng province were excluded from taking part in the study. Any persons who did not self-identify themselves as queer found in the study setting were excluded from the study. All participants less than 18 years of age were not allowed to participate in the study.

### Data collection

The study gathered data from the qualitative interviews from September to December 2023. We commenced with six pilot study interviews, which included three of each study population. This article excludes the pilot study’s findings; they enhanced the semi-structured interview guide amendments and confirmed whether the questions answered what was expected and anticipated. We sought out HCPs at their workplaces during working hours, whereas for selecting the queer participants, the principal researcher began with staff members on duty at the selected NGO clinic and then asked them to refer other queer people who visited the clinic on the day of data collection. All queer participants received a reimbursement of R250.00 for transportation.

We performed 55 interviews in the Gauteng province, South Africa, from seven district hospitals and one NGO (for public facilities), eight Johannesburg Health District offices, 20 Tshwane District Health services and five West Rand-District Health services. Participants included HCPs such as nurses, medical doctors, psychologists and social workers. The chosen study sites were district hospitals because the majority of existing research has focussed on improving primary health settings, which frequently exclude districts. We believe that district hospitals are critical for comprehensive healthcare improvements in South Africa and play a significant role in improving SRHS and dealing with the needs of queer people throughout all levels of treatment, thereby providing a more inclusive approach. We used two selected NGO clinic branches in the Gauteng province, 13 from the Pretoria branch and 10 from the Kempton Park branch. Because of the difficulties with finding queer people, we chose an available, well-known NGO that renders queer health-related services. These numbers were guided by data saturation, which means that the principal investigator stopped collecting data whenever there was no new information from either group of participants coming out. Data saturation for HCPs was achieved on the 30th interview; however, the primary investigator conducted three more interviews to validate that data saturation had been achieved. Data saturation for queer people was reached at the 19th interview session, so the principal investigator conducted three more interviews to validate the saturation. The participants were interviewed in English, yet were allowed to respond in their home languages, mostly Isizulu and Setswana, and all responses were translated into English. The principal researcher collected data with the help of one research assistant and recorded all interviews using an interview guide and a digital recorder. We explained the study’s purpose, questions and objectives to the participants prior to data collection, and those who decided to participate offered both verbal and written informed consent.

The questions asked during the data collection from HCPs and queer people focussed on the main aim, namely, to learn about the challenges both participant groups experienced. For HCPs, the questions were:

‘Tell me about some of the major challenges you have experienced during the provision of sexual and reproductive healthcare services to the queer people in your institution; how often do you render SRHS to Queer people, and what do you think are the reasons for queer people to access and utilize SRHS in your healthcare facility?’

The question for queer people was:

‘Can you tell me about your experience when you access or/and utilize the services at a public institution, and what are some of the major challenges you have experienced when you had to access or/and utilize SRHS as a queer people at a public institution?’

### Data analysis

The principal researcher employed thematic content analysis (TCA) to analyse the data, following Ravindran’s^68^ four phases. During the study, the researchers immersed themselves in the obtained data; all 55 audio files were transcribed verbatim, and wherever participants utilised and responded in their home languages, the content was translated into English. All transcripts are labelled with the interview number; for example, P1 refers to Participant Number 1 and Interview Number 1. The primary researcher analysed the data initially using *NVivo 14* based on the research questions, interview guide and interview notes, while a research supervisor served as an independent coder, and a second research supervisor reviewed the final themes as part of the article. The principal researcher and supervisor examined all the discrepancies in coding interpretations that surrounded the issues faced by both HCPs and queer people to develop a consensual viewpoint. All coded data were examined for commonalities and the emergence of important themes, which drove a subsequent thematic analysis. The *NVivo 14* analysis revealed six major and linked minor themes.

### Ethical considerations

Sefako Makgatho Health Sciences Ethics Committee (SMUREC) (Reference Number: SMUREC/H/291/2023: PG) provided ethical clearance and permission for the project. Participants in the study provided written informed consent after agreeing to participate in the study. We used pseudonyms for this work, and all comments were anonymised to ensure and maintain the confidentiality of the participants. Additionally, data are kept under lock and safe as an additional method of maintaining privacy and confidentiality.

### Trustworthiness

The principal investigator employed, followed and applied various measures to improve the trustworthiness of the study, with the purpose of increasing confirmability, dependability, transferability and credibility. For confirmability, a thorough and extensive data analysis was conducted, which included the creation of transcripts, codes, themes and repeated readings of the data. Two supervisors did peer review and independent coding to ensure dependability. Data were collected in and around seven district hospitals and one NGO clinic in South Africa’s the Gauteng province, with a clear data collection process in place to ensure transferability. Finally, for credibility, we ensured extensive data engagement through interviews, transcription, coding, analysis, continuous observation, and theme writing and summarising.

## Results

Table 1 and Table 2 depict the characteristics of the study’s participants and the list of emerging themes, respectively.

**TABLE 1:** Characteristics of study participants.

Characteristics	HCPs				Queer people			
	*N* = 33				*N* = 22			
	*n*	%	Mean	s.d.	*n*	%	Mean	s.d.
**Age, mean**	-	-	4.96	1.84	-	-	2.86	0.94
**Birth gender**								
Male	5	15.15	-	-	19	86.36	-	-
Female	28	84.85	-	-	3	13.64	-	-
**Sexual orientation**								
Straight	33	100.00	-	-	0	0.00	-	-
Lesbian	0	0.00	-	-	3	13.64	-	-
Gay	0	0.00	-	-	8	36.36	-	-
Bisexual	0	0.00	-	-	2	9.09	-	-
Transgender woman	0	0.00	-	-	7	31.82	-	-
MSM	0	0.00	-	-	2	9.09	-	-
**Marital status**								
Single	22	66.67	-	-	22	100.00	-	-
Married	11	33.33	-	-	0	0.00	-	-
**Education level**								
No formal education	0	0.00	-	-	0	0.00	-	-
Primary	0	0.00	-	-	0	0.00	-	-
Secondary	0	0.00	-	-	10	45.45	-	-
Tertiary	33	100.00	-	-	12	54.55	-	-

Straight in this study refers to heterosexuals.

HCP, healthcare professional; MSM, men who have sex with men.

**TABLE 2:** List of emerged themes.

Themes	
HCPs	Queer persons
HCPs believe queer people are afraid to consult because of embedded fears and stigma	Queer people believe HCPs have negative attitudes
HCPs as gatekeepers	HCPs as gatekeepers
HCPs are surprised by and confused about gender identity	HCPs are surprised by and confused about gender identity
Healthcare disparities and familial problems	Healthcare disparities and familial problems
HCPs’ incompetence in queer-related healthcare	HCPs’ incompetence in queer-related healthcare
Queer people as a barrier	Queer people as a barrier

HCP, healthcare professional.

The data demonstrated that HCPs and queer people in South Africa’s the Gauteng province face similar, contrasting and differing problems when rendering and receiving SRHS. Queer people typically reported unfavourable experiences with receiving SRHS in public healthcare facilities. On the other hand, HCPs conveyed their challenges with minimal positivism and acknowledged some identical challenges mentioned by queer people.

### HCPs believe queer people are afraid to seek consultation because of embedded fears and stigmatisation

The HCPs outlined that queer people were scared to visit healthcare facilities because of internalised, experienced fears and tended to distance themselves from healthcare facilities and, consequently, did not receive services. Some contrary statements emerged among HCPs, as some acknowledged that they judged queer people, and some stated that they did not.

The HCPs said queer people were afraid of being stigmatised. The participants also expressed uncertainty about why queer people did not visit healthcare facilities; however, they highlighted that queer people might have been uncomfortable with the people they would meet during visits and that because they had been stigmatised at other healthcare facilities and by community members, they believed they would be stigmatised everywhere:

‘For me I think I would say possibly some of them you find that they are uncomfortable with whoever maybe they are going to be meeting, or they are just uncomfortable with how they see themselves, then having to come and explain here. I don’t know. I’m thinking it’s got to do with them or how maybe they are being stigmatised somewhere else. I’m not sure…So, I think people- there is still a lot of stigmas in our communities and unfortunately people are afraid to come to healthcare facilities because of that, because they are going to be discriminated against and that’s something we need to fight.’ (HCP16, 30 years old)

In addition, HCPs expressed that queer people feared being judged and discriminated against. HCPs thought that some of the reasons queer people did not visit healthcare facilities were because they were afraid of being judged, rejected, criticised, discriminated against and name-called. HCPs further believed queer people worried about how they would need to look and change their identities when visiting healthcare facilities:

‘They are afraid of the judgement, I don’t know…I think what’s hindering them is that they think “Yoh I’m going to go there and I’m going to be judged” or have to change their whole outfit to identify as a person that they are not.’ (HCP19, 31 years old)

HCPs noticed that some queer people tended to withdraw and isolate themselves, which made it difficult for them to receive or be provided with a comprehensive healthcare service:

‘The ones that I got in contact with, I have not experienced any challenges but basically…they themselves tend to withdraw a little bit because I think they are scared to be free because they were lesbians, yeah!! They isolate themselves when they come to our healthcare facility.’ (HCP8, 36 years old)

Regardless of other HCPs with views and thoughts about queer people being scared, some HCPs acknowledged and agreed that they judged, discriminated and stigmatised them. This is because of the HCPs’ attitudes and inability to understand and accommodate queer people and managerial individuals in healthcare facilities by being even more judgemental about queer people:

‘So, I think because of issues such as stigma and discrimination, although we would want them to have access often or as they need like anyone else, most of them that don’t come even if it’s post sexual assault. Some of them don’t report, or they don’t even come for prep. Right? So, because of the stigma and being judged and the discrimination. But those that can actually come access family planning and condoms. And for those that maybe come through the police or the crisis centres, they access the preps. I think it’s mostly stigma, honestly, it’s mostly discrimination and I think uhm, there is still a lot of resistance to being accommodative and accepting members of the queer community, even in the healthcare sector.’ (HCP2, 36 years old)

In contrast, other HCPs indicated they did not discriminate against nor judged queer people because of the type of work they did and their professions. The HCPs indicated they treated every patient equally, regardless of their gender and sexual orientation, and would not deny healthcare services to anyone:

‘You know, as a social worker I’m working under the profession social worker. We are not allowed to judge anyone. I work with whoever gets inside the office and whatever that you mention, it doesn’t matter whether you are lesbian, whether you are gay, remember, that part of individualising people and respecting what the patient or the client wants stands out. I cannot say no because you are a lesbian, I cannot help you.’ (HCP13, 55 years old)

### Queer people believe HCPs have negative attitudes

Queer people felt HCPs disapproved and resented them by making harsh comments and jokes, and by gossiping. In addition, queer people held that HCPs were judgemental, stigmatising and discriminating towards them, and, as a result, they isolated themselves from public healthcare facilities.

Queer people reflected on their experiences at public healthcare facilities. Some preferred NGOs because of the safety and love they felt there, whereas others indicated that some HCPs judged them and questioned them about why they chose to be homosexuals. As a result, they felt sadness and hated themselves:

‘Sometimes they are judgmental, they call us with names, and it makes me feel so bad, just imagine someone who supposed to help you is the one judging you, one gets bored already.’ (Transgender woman, 25 years old)

‘I’ll rather go to an organisation like this and not do it publicly, I come here and do my things because they understand me. But going to a public you get judged, yes, and especially when you ask- like come and talking to a nurse and ask you about your sexuality and you tell them your sexuality then they would come like nurses would be like how come you be gay, this is that. They have those judgmental thoughts, they’ll give you that and they’ll make you like leave that room hating yourself, yes. That’s why I would rather prefer coming to places like this and just do my thing so that no one is going to judge me, all of us we’re all the same.’ (Bisexual, 32 years old)

Queer people highlighted that some HCPs were homophobic, stigmatising, nosy and curious once they noticed you were a member of the LGBT community. The queer participants stated that the HCPs would go to the extreme of calling others to gossip or asking too personal questions, such as how they had sexual intercourse. The queer participants further indicated that they deserved respect and the same treatment as anyone else visiting public healthcare facilities:

‘Uhm, some nurses are homophobic obviously so as soon as you get there, sometimes you go there just to test and maybe or you have something like that is not okay with you and then they will now start asking you uhm are you a girl, are you a what, and then you start to say I’m a transgender, transgender what, what’s that and whatsoever, according to my own experience that it has happened to me. So, now I believe that uhm I expect most of all respect and equality with everyone, yah.’ (Transgender woman, 25 years old)

The queer participants indicated that one of the reasons they isolated themselves was because they were not accepted in public healthcare facilities. Isolation was one of their coping mechanisms, protecting their space so that the HCPs’ stigmatisation did not affect them:

‘You can’t relate to people who do not like you, then one tries to like to have this uhm I don’t know if I can say like a coping mechanism for themselves whereby, I say okay this is where and who I am and then I distance myself and not say much to create this working mechanism so that I cannot be affected through the whole stigma outside, actually.’ (Gay, 27 years old)

### HCPs as gatekeepers: HCPs’ experiences

Based on the HCPs’ responses during the interview sessions, the study noted that they continued acting as gatekeepers and hindered queer people from accessing and utilising SRHS in public healthcare facilities. This theme emerged from the HCPs’ responses.

The majority of HCPs stated they assumed the gender orientation of queer people just by looking at them as they walked into their consultation rooms and healthcare facilities. As a result, they anticipated that queer people would not require certain services and some linked queer people with particular illnesses such as HIV. Other HCPs classified queer people as looking normal, which then caused them to generalise patient care:

‘When you see them walk in, automatically you click your mind that what is she coming to do hear (sic) at ANC clinic, she can’t be pregnant, she is butch; but she is pregnant or he is gay he is probably HIV positive, you know those, yah.’ (HCP11, 25 years old)

‘Okay, Iyo! It is difficult hey, some other time I saw lesbian client and I wasn’t aware because she is normal, but luckily, she came with the mother. They were reporting sexual assault but when I ask if she is sexually active, she then told me that it was the first because she is a lesbian and I was like shocked also not knowing how handle it, I didn’t know what to say and do further.’ (HCP30, 37 years old)

Some HCPs believed that their own religious beliefs and cultural practices were barriers to queer people coming forward to seek SRHS. They further stated that they were still cultural and religious beings, which, ultimately, made it difficult for them to seek advice on other services:

‘Eish our cultures, like culturally it’s still a taboo, we say how can you get an egg from someone then the ancestors whatsoever, you understand. If now you are getting a strange person’s egg, but this child is going to be yours, those kinds of things. So, culture is still one of the things that come in the way that, I mean, interrupts a process that a lot of people are willing and open to take but now afraid of the culture and the belief system that now stands in the way, it then becomes difficult for lesbians to access IVFs freely.’ (HCP21, 43 years old)

A lack of interest and HCPs being uncomfortable with talking and rendering services to queer people were observed during the interview sessions. One HCP conveyed that they were not sure what types of services they should render to queer people, and they were not even capable of conducting physical assessments of them:

‘I am not sure if they come what I should screen… because let’s say they are presenting with an STI right, Hai, I don’t think I will be comfortable and ready like imagine why would I check their anus? I don’t want to see any trauma there, any legions (sic), any, you know but I don’t think that’s like number one.’ (HCP11, 25 years old)

### HCPs as gatekeepers: Queer people’s experiences

Some observations were obtained during interviews with queer people, related to HCPs indeed being gatekeepers resulting in them not being interested in accessing SRHS. In addition, the queer participants stated that HCPs assumed their gender based on how they appeared; hence, they reported negative attitudes and treatment by HCPs. However, some queer people stated they had never experienced challenges at all.

A significant number of queer people pointed out that HCPs did not ask them about their gender because they just assumed it, leading to the wrong services being rendered and offered. Other queer people stated that HCPs treated them like women without asking what they preferred to be called, as this would help the HCPs to identify their sexual behaviours:

‘I don’t know if I should say I was scared, embarrassed or traumatised, I was at the government clinic and a nurse asked me for my pee, you understand, then I went and pee and then when I came back– so I thought maybe she was just testing acidity level in my urine only to find out that she was doing a pregnancy test. Oh well she was not aware that I’m not female, she assumed that I was female. So, she did a pregnancy test and then while waiting for ama-results, she asks me when last did I go to my periods, then I’m like no I don’t get periods, I’m not a girl.’ (Transgender woman, 30 years old)

‘Ai with me, they treated me as a woman, yes I guess it’s that– the only time I felt, I guess, sort of unsafe, I had a girlfriend I think, the first time I had a girlfriend I think I was 17 towards 18, and that’s when I said that was the only time I’ve had an STI and I got that because she was using things that are not yah and I went there and I couldn’t explain why because first of all I’m not having sex with boys, why do I have because she concluded.’ (Lesbian, 28 years old)

The queer individuals received negative experiences, criticism and disapproval from HCPs whenever they visited public healthcare facilities. A substantial majority of queer people highlighted that they had unpleasant experiences, felt uncomfortable, were forced to undergo some procedures and HCPs tried to convince them to become pregnant:

‘My experience was bad, So, what happened is that I got there and then obviously the admin was doing whatsoever, you give them your ID and then after that I was sitting. So, as I was about to go in, I went in– okay not before, the nurse came and checked my file and then he was like you are not a male, but your file says you are a male, and then she looked at me and be like oh you’re one of and then she left me there. And she went inside, the other nurses came out and then they started looking at me and then are like oh okay [Indistinct] and whatsoever. So that was very uncomfortable because of I had to leave right there.’ (Transgender woman, 25 years old)

Regardless of the majority of queer people experiencing all sorts of challenges when accessing SRHS, some stated that their experience was good, and they had no challenges at all. Queer people further praised HCPs for their work. Some queer people reflected they had never experienced challenges because they did not go around talking about their sexuality, while others did not look like their sexual identity:

‘Honestly, I don’t think it was challenges, I don’t think we had any challenges. Doctors and nurses are trying to help.’ (Gay, 24 years old)

‘I don’t have. I’ve never experienced any stigma or any hate speech or anything. The thing is I’ve never preached, or I’ve never came out to tell people that hey I’m gay. You see, I think my surrounding, even my family knows that my mom, I’ve never explained to my mom or my parents that I’m gay.’ (Gay, 30 years old)

### HCPs being surprised and confused about gender identity: HCPs’ deliberations

The HCPs acknowledged that they still did not understand queer identity and that they would continue to ask questions because they were not used to such identities. Consequently, this might be one of the factors causing queer people not to visit healthcare facilities as they feel uncomfortable when asked for clarity and reasons for their gender identities:

‘I think, Yoh… the biggest challenge I have seen was a lady, she came to deliver, I was working in labour ward, she came to deliver, a lady, now this lady came to deliver, when she gets discharged, she is fetched by another lady, and they say this is the father to the baby, but this is a woman. You can see this is a woman, why are they saying this is the father to the baby. They saw my expression and being shocked and they might feel bad and not come back.’ (HCP11, 25 years old)

‘Ee, only. But when I ask you are you a female or a male, oh wow, oh okay. You will see even their facial expression changing and some of us, remember we are human beings, we are still going to be surprised and be shocked, we will even want to give you counselling as in why are you gay, why are you lesbian? You understand? They still get those questions even if we can hide it, even if… for me I think it’s discouraging for them to say I’m still going to be asked those kinds of questions, so I am not going to go and get the service that I need.’ (HCP13, 42 years old)

### HCPs are surprised and confused about gender identity: Queer people’s deliberations

The queer participants stated they had observed HCPs being shocked and surprised whenever they were themselves and presented as queer. This has been one of the barriers for queer people to access and utilise public healthcare facilities freely. They further stated that HCPs asked too many personal questions, encouraging them to resort to private doctors:

‘Yah, so most of the time like when I have health issues– maybe when I seek medical attention, I just go to like a private doctor, just like a doctor yah, because I remember I once went to a clinic, its government clinic so when they found out that I was gay, they were surprised than a handsome man like me would be gay, they started asking me how I have sex and decided to test me HIV without counselling, I then decided I will never come back again here.’ (Gay, 24 years old)

### Healthcare disparities and familial problems: HCPs’ discussions

HCPs mentioned that some of the challenges inhibiting queer people’s access and utilisation of SRHS were because their healthcare facilities were not inclusive. Moreover, other HCPs stated that healthcare facilities had limited resources, which might place them at risk of acquiring infections and lack regulations for registering a child of the same gender. Some HCPs remarked that reproductive services were expensive for queer people and, overall, healthcare facilities with exclusive areas for queer people, such as male and female toilets and a lack of privacy:

‘And there are limited resources for them because I’m trying to think if it’s two females, then they need those resources to be intimate with. They use their own money. It’s not available in government hospitals because people will judge you. Why do you want that toy? Because we think it’s too females and then. What? So those resources they use are not available? Yes! So, they need to have money to purchase them. That is why they end up maybe doing wrong things now they end up having infections because they can access whatever resources because they are not cheap as well.’ (HCP1, 40 years old)

‘The major challenge is our government hospitals, I don’t think they cater them well, reason being if you look at our toilets it’s labeled male, it’s labelled females. There is she coming, it’s a shemale coming to the hospital, feels I’m a man, where does she supposed to go now; there’s a toilet labeled male, there’s a toilet labeled female.’ (HCP22, 42 years old)

A lack of support from family and relatives was pointed out as one of the reasons making it difficult for queer people to access SRHS, regardless of HCPs’ efforts to assist them. In addition, other HCPs highlighted that family members found it difficult to accept and support their queer children but were accepted by other community members:

‘You know, the only challenge that we have, which I think maybe it becomes a hindering fact of working with an individual, when the family doesn’t want to accept their child. Yah, it’s because of the sexuality and of the adoption matter.’ (HCP13, 42 years old)

‘I have only encountered one gay patient. It was long ago. The challenge now was based on that the community– him being– him identifying as a gay person, it was challenging for him because he was now admitted because at home, they also cannot accept him, you know, so it led him to such an emotional distress that he ended up being admitted. it was disheartening to see that. But he was only having challenges with his family, but it was– what I have noticed is that the community has already accepted him.’ (HCP 28, 27 years old)

The HCPs’ observations were that queer people were being abused and discriminated against by other community members. Further, other HCPs acknowledged that awareness campaigns needed to be launched with community members for them to understand that queer people were like any other person and needed support:

‘For me right now, as a social worker, when it comes to sexual reproductive health for queer people, I think that’s the one thing that I can think of. They want kids, you know, or maybe it’s the issues as well. We face many issues of the LGBTQI communities being abused, a lot, right? So, maybe it might be that; “I have been raped”; “I have been abused on” wherever, at home, or “I’m being discriminated against.” That is why I’m here as well, as a social worker to be able to offer that support to say okay, what do you need? How can I give you that support so that we report the abuse that’s happening?’ (HCP28, 27 years old)

### Healthcare disparities and familial problems: Queers’ discussions

Queer people believed public healthcare facilities lacked sexual reproductive services because of affordability. Others mentioned delayed services and that they were excluded by being called special cases:

‘I feel that we are just not being prioritised like other straight people, you know, or maybe is it because we are looking for a specific thing, I’m trans and I need medical attention, this is what I want. Unfortunately, there are limited services to cater us at the public hospitals and clinics, yes.’ (Transgender woman, 20 years old)

‘Yoh, I needed IVF or what do you call IUI, it’s like 50K to do IUI or IVF, even that 50K doesn’t even include consultations, doesn’t include blood work, doesn’t include scans, doesn’t even include sperm donor, you know what I mean, I still need all of that. Meaning at the end of it all I would have spent more or less 100K.’ (Lesbian, 30 years old)

Similar to what HCPs observed, the queer people indicated that they tended to be abused and discriminated against by other community members and were accepted by their family members:

‘Firstly, coming out as gay, you know, people will be like Yoh, I don’t want to talk to this one, you know, name-calling, you know those offensive names man. So that’s the first challenge we faced, most of us, in the communities. You are even scared of coming to the clinic because the community members will judge you. So– okay, family [-] wise it was fine because they’ve long accepted me because I think the parents saw from an early age.’ (Transgender woman, 23 years old)

### HCPs’ incompetence with queer-related healthcare: HCPs’ interactions

The study observed a lack of skills, knowledge, experience and expertise regarding queer-related health matters among HCPs. Most HCPs showed that when rendering SRHS to queer people, they were too empathetic, felt uncomfortable or did not know how to deal with queer people without making them feel judged and discriminated against. They acknowledged that they were not trained in dealing with gender-diverse patients and some stated that they did not know what types of services to render to queer people.

HCPs’ empathy seemed to prevent them from further probing during history-taking when rendering various services to queer people:

‘I think it’s probably myself where I will be wondering should I ask for details, or should I ask for details or should I ask for this and that. I think it is a question that I will have with myself, and I would always be battling with that. Is it important– the detail– or is it not important, because sometimes you may want to ask a question, but it’s not necessary, so, for me it’s always to ask myself that question, do I have to ask this question or not, or am I just asking out of curiosity? So, it is always to know, and sometimes it is not easy to know whether you can or you can’t; so, I would say that is the issue that I have to always ask myself do I have to ask a question or what I have is enough.’ (HCP5, 47 years old)

Some HCPs appeared uncomfortable with asking queer people questions; they were concerned about not appearing rude:

‘The approach. I feel that like if– it will be rude for me just to come up to a person and ask about their sexuality and on how do you have sex, how do I start, or just to ask what is rimming, it’s very difficult I would say, yah.’ (HCP18, 32 years old)

‘For me personally I am not working in the department but when I go there, I also honestly felt a little bit of that the uncomfortable, yah, you will feel a little bit discomfort, you know, yah so I think that’s one of the… It’s actually the only challenge that I can think of right now, yah.’ (HCP9, 57 years old)

Not knowing how to address queer people without making them feel discriminated against was one of the HCPs’ challenges, which showed that HCPs indeed lacked skills and expertise in handling queer people:

‘I can’t say their pronouns, I mean that’s something– sometimes it makes us feel– me specifically, I can’t say shy but sometimes I feel like I can’t, I don’t know how to address them, can I say ma’am or sir. Some patients can feel offended, this person just because I’m a male and I have a makeup on it doesn’t necessarily mean you can say– like think that I’m gay or what, maybe I just like wearing this and you come to me think now because oh, a lady, I’m still a sir. Hai, I don’t really know, and I have no information.’ (HCP32, 28 years old)

Some HCPs were ineffectual and did not know what they should offer queer people seeking SRHS. Others were uneasy about how their sexual practices might cause health challenges but still did not know how to prevent those health issues from occurring. The HCPs continued stating that it was difficult to render SRHS to queer people, yet they continued treating them like any other patient visiting their healthcare facilities:

‘And then again, regarding their health, remember they practise anal sex, so I think at some point there’s a stage where their muscles loosen, and then they develop some illnesses, I think so, yes. They develop some illnesses. So, I don’t know how we can stop that, how we can prevent that from happening. Hai!! I don’t know.’ (HCP10, 36 years old)

‘I am not even sure, Yoh, that one is a bit difficult because I’m not sure; like if they are transgender, someone who’s a transgender…which is quite tricky when it comes to the queer, LGBTQI community because now when you are dealing with trans individuals, whether a trans man or a trans woman, it’s a whole different ball game, you know. So, yah, then there’s that overlap in between that is sometimes a bit difficult to navigate.’ (HCP16, 30 years old)

A lack of training continues to be a significant issue for HCPs, which includes training on queer-related matters. The majority of HCPs acknowledged they did not have skills and knowledge about queer people’s issues, while some HCPs stated they did not know how to care for or render services to queer people because their tertiary curriculum covered only male and female content, not queer people’s healthcare:

‘I think the other, the other one, is also lack of training actually, amongst the healthcare workers regarding you know how the use of words, the use of correct pronouns, they use, how to treat, you know how to be cautious and caution, yeah, cautious, and also how to be uhm not accommodative, but to actually treats people as they are. So, there is also that I think you know some healthcare workers struggle with, you know, the use of words, understanding some people still consider members of the queer communities as outsiders as attention seeking, as some people who are who have trained in all schools still consider it some kind of a medical condition, something they need to convince someone and treat or so I think what I can think of in terms of challenges is the stigma and discrimination.’ (HCP2, 36 years old)

‘The thing is the course that we did is only the female part, but the LGB, the others, we were not involved especially when it comes… because most of my patients, especially males would ask “Sister, why not us, only females?” And I will tell them I do not have any clue or any information or any workshops or any teachings regarding what to be done or how am I going to help you.’ (HCP6, 42 years old)

### HCPs’ incompetency in queer-related healthcare: Queer people’s interactions

The queer participants highlighted that HCPs asked about sexual activities once they noticed they were members of the queer community. They stopped focussing on the reason why queer people came to the clinic in the first place and dwelled on who they had sex with and how they did it:

‘Yah, I remember I was raped, and they took me to the government hospital, and you know that doctor there, the questions he was asking– and he was male, the questions he was asking were very insensitive. So, like okay so the docket says you were raped so this is what I’m going to do to you. Like he explained the whole process and everything. Yoh, and while doing that, questions he was asking like so is it painful, you know, like weird questions. And so how is it, is– now he’s no longer on the rape case, now his asking me on a personal level, so like tell me how it feels to have anal sex?’ (Transgender, 30 years old)

The queer participants reflected that HCPs asked uncomfortable and overly personal, invasive questions, leading to queer people lying about their sexuality. Sometimes, queer people were unable to answer and be free about who truly they were when answering the questions asked by HCPs because of a fear of judgement. Furthermore, the queer people indicated that HCPs did not understand what queer people went through, which made it difficult for them even to explain further:

‘Nurses ask too personal questions, for example maybe you have an STI, they ask questions like uhm who are you sleeping with and it becomes difficult like to say oh I’m having sex with a boy because you’re scared of– even if they’re not going to say it but the looks cause probably there’s going to be like maybe another nurse there and whatever but like the looks and whatever now you have to explain how you guys have sex which I didn’t come here for that, I just uhm I came here for– to seek medical attention not to be– understand. So, at the clinics I think the most challenging thing is having to explain to people who don’t really understand, yah they just don’t understand and yah. Some of the questions are quite invasive, yah, so you end up lying even like most of the time I’ll just say I have a girlfriend, yah.’ (Gay, 24 years old)

The study noted HCPs’ refusal to provide services to queer people. This might be because HCPs still do not understand the importance of what SRHS for queer people could do in terms of protecting them and preventing many illnesses.

‘I went to the clinic to ask about PrEP because of it was new at that time, they were like no we cannot give you a PrEP, you’re still young and stuff like that. I was like okay, and I was sad that I cannot protect myself from sexual transmitted infections.’ (Gay, 27 years old)

### Queer people as a barrier: HCPs’ discourse

The vast majority of HCPs indicated that even though they were labelled gatekeepers, they lacked skills and knowledge about queer-related matters, and queer people themselves were the obstacles to receiving a comprehensive healthcare service. They stated that queer people were unable to speak freely to them and felt that they should always explain themselves to HCPs regarding their sexualities. In addition, the HCPs mentioned that queer people utilised the private sector as they felt safer there, and HCPs in the private sector were held accountable if they treated patients badly. Lastly, various groups of HCPs indicated that queer people were not comfortable during examinations, and some queer people did not return for follow-up care:

‘Then with others they don’t want to be or like to be undress, looking at them. Others don’t want at all, Yah I think so because it’s like, according to my understanding, like what they are now is what, now they are boys right … you grew up, you changed to be a girl, and then it’s not simple for you to undress for each and every person.’ (HCP12, 55 years old)

‘You know, what I think is happening now is that it’s becoming a disclaimer, you know. It’s like okay this patient is coming with a disclaimer, they are transgender, disclaimer this and that, and I think that adds to that, you know, but at the same time I think the idea behind putting the disclaimer is to be sensitive and might make them feel uncomfortable to come to the facilities.’ (HCP17, 24 years old)

### Queer people as a barrier: Queers’ discourses

Lastly, the data highlighted how difficult it was being a queer person, which led to many queer people changing how they behaved and appeared whenever visiting public healthcare facilities. This was because they were afraid to be judged, thus affecting the services they received because HCPs treated them like heterosexuals. Some queer people stated that not all public clinics and HCPs treated them with an attitude, as they felt uncomfortable themselves when talking about their sexuality. Other queer people, specifically transgender individuals, confirmed what HCPs had said, namely that they did not want to undress, stating that they felt uncomfortable being treated by male HCPs. In addition, they indicated that female HCPs also felt uncomfortable attending to them:

‘I wouldn’t say the local clinic back at home was the nicest place to go to for PrEP but then yah I– yah, yah, that’s it, I was just not comfortable. They even asked me questions like “why are you taking PrEP” and I had to lie that you know I have a girlfriend in Pretoria, that time I didn’t have a girlfriend in Pretoria but then it was not the space for me to say no I’m having sex with men in Pretoria so–.’ (Bisexual, 21 years old)

‘I would feel uncomfortable to be attended by a male nurse, remember I have to take i-treatment of STI sometimes I need to undress so that I can get the shot, so I feel uncomfortable being attended by a male nurse and at the very same time female nurses are also uncomfortable attending us sometimes, I’m not saying always but sometimes.’ (Transgender woman, 30 years old)

## Discussion

Our findings show that HCPs detect a common sense of anxiety among queer people seeking healthcare services, which is consistent with previous research indicating that queer people have mental health difficulties like anxiety.^46,69^ Further, a prior study found that homophobic reactions were substantially related to anxiety and sadness, linked to stigmatisation or discrimination in employment settings and healthcare institutions.^64^ Given these findings, it is imperative that healthcare facilities and sectors create safe spaces and inclusive environments for queer people so that their anxieties and fears can be alleviated. This approach will help prevent stigmatisation and discrimination against queer people when seeking healthcare services and in their workplace settings.

The HCPs explained that internalised, experienced concerns frequently cause queer people to avoid healthcare facilities, with concerns ranging from potential judgement to stigmatisation. Researchers agree that queer people have reported discrimination, distrust and terror in hospital settings.^30,44,70^ Contrary views within the HCP community demonstrate a lack of consensus, with some acknowledging judgement of and prejudice against queer people, while others claim to treat all patients equally, regardless of gender or sexual orientation. Taking these insights into account, it is important that frequent training programmes and sessions for HCPs regarding inclusivity, the negative impact of their stigma on queer people in relation to healthcare services and the utilisation of those who are treating queer equally as champions to influence the ones who are still having challenges should be introduced at different healthcare facilities.

Our study adds to the synthesis of available knowledge by Beagan et al.,^71^ who found that the majority of participants believed there were no substantial disparities between primary care for queer women and care for all other patients. This indicates that sexual orientation and gender identity were widely regarded as unimportant to care provisioning, as HCPs, such as physicians, treated everyone the same.^71^ Furthermore, they underlined that they constantly endeavoured to suspend any prejudices, remained nonjudgemental with all patients and avoided becoming distracted by aspects of a patient that the physicians themselves might find problematic.^71^ However, Seretlo and Mokgatle^48^ found that some primary healthcare (PHC) nurses had a judgemental attitude and were surprised by queer patients – associating their sexuality with childhood traumas – while other PHC nurses were nonjudgemental and willing to serve patients despite their sexuality. In addition, a Tanzanian study found that some HCPs actively limited access to healthcare for men of various genders and sexualities.^72^ With these findings in mind, it is critical that all HCPs require training on how important every person is regardless of their sexuality and gender. Government and policymakers could develop robust policies, guidelines and procedures to guide HCPs so that can be utilised when caring for and managing queer people thus ensuring and achieving synthesising of care and minimisation of judgemental care.

According to our research, queer people are concerned about being stigmatised in healthcare settings and fear judgement, criticism and discrimination. Again, research findings highlight a significant disparity in HCPs’ opinions regarding queer people: while some admit to making judgements and discriminating against patients, others insist on treating everyone equally. However, queer people regularly reported unfavourable encounters with healthcare providers, such as rejection, hostility and inappropriate probing. These experiences contribute to queer people’s unwillingness to use public healthcare facilities, and instead preferring NGOs, where they feel safer and more accepted. Our discussion is framed within the context of multiple studies suggesting that participants, for example, indicated that HCPs told every colleague in the healthcare facility about them and that they were humiliated, with some HCPs even seducing them.^73^ According to one study conducted among Durban University students, HCPs continue to be prejudiced towards homosexual persons and define HIV illness as a gay disease.^74^ From different angles, researchers in the field have consistently observed that the percentage of primary care providers (PCPs) with negative sentiments about queer people differed significantly among studies in the United States of America.^75^ In Tanzania, some HCPs saw sexual activities of MSM as aberrant and referred to them as ‘foolish guys’.^76^ Considering these outcomes, it is essential for policymakers, managers and HCPs to prioritise inclusiveness. This can be achieved by installing notice boards that indicate the types of services available for queer individuals in healthcare facilities. Such measures may change the perceptions that queer people have of healthcare settings and improve the uptake of SRH services without fear. Additionally, it is crucial for professional bodies to be involved in the introduction of legislation that mandates HCPs to ensure fair and equal access to SRHS for queer people. These legislations could influence the training offered at higher education institutions to promote inclusive practices concerning queer-related matters and enhance cultural competency.

The participants expressed unease with the persons they could encounter in healthcare facilities, often as a result of previous experiences with stigmatisation in both hospital settings. These findings are consistent with the findings of Moagi et al.,^46^ who found that stigma and prejudice hampered the health and well-being of sexually and gender-diverse people. Furthermore, our findings are consistent with those reported by Ross and Setchell,^53^ who found that some HCPs identified discrimination as a theme, which included both obvious and subtle forms of discrimination, as well as concerns about discrimination within the larger community. Similarly, Dean et al.^27^ state that queer groups have considerable obstacles in accessing sufficient healthcare because of societal stigmatisation. Concerns about how they would be viewed, as well as the possibility of having to change their sexual identity to obtain healthcare services, were recognised as significant impediments for queer people. These observations underscore the need for government and policymakers to put into practical actions and activities to prevent queer people’s stigmatisation such as mandatory training for HCPs on queer, gender-affirming policies, queer-friendly signs within the healthcare facilities and sufficient allocation of queer resources such as lubricants, hormonal, adoption and other relevant services. Furthermore, the promotion of queer health-related issues awareness within the healthcare facilities and collaboration with existing queer NGOs to enhance knowledge, skills and expertise of HCPs can also be considered.

Our findings also demonstrated that HCPs frequently operate as gatekeepers by assuming queer individuals’ gender orientation based on their appearance. This notion, along with cultural and religious beliefs, impedes access to SRHS. The dearth of inclusive healthcare facilities, as well as familial non-support, have been noted as significant challenges for queer people, resulting in healthcare disparities. Our findings are consistent with a South African study that found that HCPs attributed their moralising, judgemental and homophobic attitudes to their Christian beliefs and ideals.^73^ Again, some claim that providing healthcare services to people in same-sex partnerships is difficult because they do not believe in homosexuality.^73^ These studies demonstrate that religious beliefs, cultural traditions and values impact HCPs’ views about homosexuality and how queer people are treated around the world. Based on these results, it is crucial again that policymakers, government stakeholders and private stakeholders hold hands together to develop and implement activities that will enhance professional ethics and queer-centred care regardless of HCPs’ beliefs.

In terms of HCPs’ competence, our research reveals a significant shortage of skills, knowledge and training in queer healthcare. Further, HCPs’ empathy, while typically positive, may impede effective communication and service delivery to queer people, highlighting a need for increased education and cultural competence within healthcare institutions. In synthesising results from multiple studies, we can conclude that one of the most significant barriers to utilisation and accessibility of healthcare services for queer people is a lack of education, understanding and competence among HCPs regarding queer people’s health-related issues.^44,47,77^ Our findings are consistent with several studies that found HCPs’ absence of awareness about transgender-related health issues,^60^ as well as about the unique needs of this community and being insensitive about those^61^ and a lack of understanding of issues specific to transgender well-being.^53^ In light of these discoveries, it is necessary that all government and private healthcare higher education institutions develop comprehensive queer health curricula thus ensuring that HCPs are well equipped with queer-related foundational knowledge, skills and expertise. Moreover, this calls for the establishment of ongoing professional development opportunities, such as in-service training, workshops, mentorship and peer support networks for HCPs to share their challenges, gain potential solutions and provide equitable care for the queer community.

This study also found that HCPs were confused about queer people’s gender identities, which caused discomfort and hampered access to treatment. On the other hand, queer people reported being treated unfairly and asked intrusive questions and encountering difficulties during physical examinations by healthcare providers. This supports the conclusions by Mirza and Rooney^29^ that discrimination in healthcare settings endangers queer people’s lives through delays or the denial of medically necessary care and discourages queer people from seeking healthcare. Although South African government has an online tool for HCPs called knowledge hub which helps HCPs to provide effective care for every patient within the country, it is vital that more innovative technological tools be introduced to enhance their skills whenever confused about what type of care to render to the queer people. Additionally, policymakers could utilise the existing tool to add queer-related matters in the form of modules, webinars, simulations and forums thus improving access and provision of SRH to queer people.

## Study’s strengths and limitations

Our study provides a comprehensive picture of the issues queer people and HCPs face. Because we included various participant categories and locations, our study produced more representative information from both parties. Moreover, our study improves transferability because the findings are more likely to be applied to a broader context within the same province. The final strength of our study is the dependability of the results, as we observed similar patterns and responses from both participant groups, supporting the study’s trustworthiness and credibility. There were some limitations during data collection as some queer people were underrepresented, such as lesbians, intersex persons, bisexuals and MSM.

## Conclusion

Finally, our study’s findings illuminate the variety of obstacles queer people face when accessing healthcare services. Further, the findings emphasise the critical need for increased education, training and cultural competence within healthcare systems to eliminate current obstacles. Lastly, the study advocates for inclusive and respectful healthcare for all people, regardless of sexual orientation or gender identity.

## Recommendations

Based on the study’s findings, healthcare organisations should adopt extensive education and training programmes focussing on cultural competency. These initiatives should attempt to break down current barriers by creating an inclusive and respectful atmosphere for everyone, regardless of their sexual orientation or gender identification. This strategy will presumably ensure equal access to healthcare services and enhance overall wellness for queer people.

In addition, tailored information sessions should be held for both HCPs and queer people. These sessions should enhance HCPs’ education and sensitivity to queer health issues, preparing them to deliver respectful and educated care. Queer people should attend sessions that educate them about their healthcare rights, available services and how to navigate the healthcare system efficiently. This dual strategy would serve to eliminate the divisions between queer people and healthcare providers, promoting trust and understanding between them.

Lastly, developing innovative healthcare solutions to address SRHS for queer people could help reduce disparities in healthcare and eliminate the challenges faced by both HCPs and the queer community.
